# Inhibition of NA^+^/H^+^ Exchanger 1 Attenuates Renal Dysfunction Induced by Advanced Glycation End Products in Rats

**DOI:** 10.1155/2016/1802036

**Published:** 2015-11-30

**Authors:** Peng Li, Geng-Rong Chen, Fu Wang, Ping Xu, Li-Ying Liu, Ya-Ling Yin, Shuang-Xi Wang

**Affiliations:** ^1^College of Pharmacy, Xinxiang Medical University, Xinxiang 453003, China; ^2^Department of Pharmacology, Pharmaceutical College, Central South University, Changsha 410078, China; ^3^The Key Laboratory of Cardiovascular Remodeling and Function Research, Chinese Ministry of Education and Chinese Ministry of Health, Qilu Hospital, School of Medicine, Shandong University, Jinan 250012, China; ^4^Medical College of San-Quan, Xinxiang Medical University, Xinxiang 453003, China; ^5^School of Basic Medical Sciences, Xinxiang Medical University, Xinxiang 453003, China

## Abstract

It has been recognized that sodium hydrogen exchanger 1 (NHE1) is involved in the development of diabetic nephropathy. The role of NHE1 in kidney dysfunction induced by advanced glycation end products (AGEs) remains unknown. Renal damage was induced by AGEs via tail vein injections in rats. Function and morphology of kidney were determined. Compared to vehicle- or BSA-treated rats, AGEs caused abnormalities of kidney structures and functions in rats, accompanied with higher MDA level and lower GSH content. Gene expressions of NHE1 gene and TGF-*β*1 in the renal cortex and urine were also increased in AGEs-injected rats. Importantly, all these detrimental effects induced by AGEs were reversed by inhibition of NHE1 or suppression of oxidative stress. These pieces of data demonstrated that AGEs may activate NHE1 to induce renal damage, which is related to TGF-*β*1.

## 1. Introduction

Diabetic nephropathy is one of the most important complications in diabetes and is also the leading cause of renal failure in adults. AGEs are a heterogeneous group of products in which protein and lipids are covalently bound to sugar residues under hyperglycemic and oxidative stress situations, which is proposed to play a major role in the pathogenesis of diabetic nephropathy [1]. However, the mechanisms involving the pathogenesis of renal damage induced by AGEs were poorly understood.

The Na^+^/H^+^ exchanger (NHE) is a protein that is expressed in many mammalian cell types [2], exchanging one intracellular H^+^ for an extracellular Na^+^. In this way, it regulates intracellular pH value and cell volume [3]. To date, nine isoforms (NHE1-9) have been identified. NHE1 is ubiquitously distributed in most tissues, which is localized in the membrane and sensitive to amiloride. It is involved in signaling transduction and regulation of cell functions [4]. Our previous studies have indicated that hyperactivity of NHE1 exchanger is related to the vascular injury associated with high glucose or hyperglycemia [5–7]. Cariporide, similar to amiloride, as a selective NHE1 inhibitor, prevents the process of vasculopathy in diabetic rats [8, 9]. Although its role in diabetic vascular complication has been extensively investigated, whether NHE1 mediates diabetic nephropathy and the pathogenic mechanism remain unclear.

It has been reported that AGEs-induced hyperglycemic memory phenomenon [10–12] is very similar to the persistent NHE1 activation in diabetic nephropathy [13]. These findings suggest that the mass accumulation of AGEs may involve activation of NHE1 in the pathogenesis of nephropathy. Our previous studies have also demonstrated that AGEs activate NHE1 to induce proliferation of vascular smooth muscle cell [14]. Therefore, we hypothesized that activation of NHE1 may be a critical step in the signal transduction of AGEs-induced renal damage. Our results demonstrate that cariporide, via inhibition of NHE1, normalized the redox status to protect renal function in rats injected with AGEs.

## 2. Materials and Methods

### 2.1. Materials

Cariporide, N-acetylcysteine (NAC), Bovine serum albumin (BSA, cat. A1933, reagent ≥ 98%), and D-glucose were purchased from Sigma Company. Antibody to AGEs receptor (Ab-RAGE) was purchase from Santa Cruz Company. BCA protein assay kit was brought from PIECE Company.

### 2.2. Animals

Male Sprague-Dawley rats (8 ± 2 weeks old, 180 ± 20 g) were purchased from the Center of Experiment Animals, Central South University (Changsha, China). All rats were housed individually in cages at a room temperature of 21 ± 1°C with a 12 h light/dark cycle and were given free access to food and water. At the end of the experiments, rats were placed in individual metabolic cages and 24-hour urine samples for three consecutive days before sacrifice were collected. After fasting for 12 h, the rats in each group were anesthetized with sodium pentobarbitone (30 mg/kg, I.P.) and exsanguinated. The right kidneys were collected after perfusion with 40 mL of ice-cold PBS and stored at −80°C. This study was carried out in strict accordance with the recommendations in the Guide for the Care and Use of Laboratory Animals of the National Institutes of Health. The protocol was approved by the Committee on the Ethics of Animal Experiments of the University of Central South.

### 2.3. Preparation of AGEs

AGEs were prepared* in vitro* as described previously [14]. Briefly, BSA (50 mg/mL) was incubated with D-glucose (0.5 M) in PBS supplemented with penicillin (100 U/L) and streptomycin (100 mg/L) for 12 weeks at 37°C under sterile environments and darkness. After incubation, the solutions were dialyzed against PBS (pH 7.4) at 4°C for 48 h to remove free glucose, following separation of AGEs into aliquots, and stored at −20°C. The protein concentration was measured with the method of BCA. AGE-specific fluorescence was determined using 370 nm excitation and 440 nm emission wavelengths by using a spectrofluorometer (Shimadzu, Beijing Beyond Technology Development Co). BSA was incubated in the same conditions without D-glucose and served as control of AGEs.

### 2.4. Preparation of Renal Slices

As described in details previously [15], the isolated kidneys were immediately placed in 5 mL ice-cold Krebs buffer and kept on ice. The slices were rinsed two times in 5 mL oxygenated Krebs buffer each for 3 min at 25°C in an oxygen environment with constant shaking and then transferred to 3 mL oxygenated Krebs in designated Erlenmeyer flasks and equilibrated for 10 min at 37°C prior to different treatments.

### 2.5. Examinations of Renal Function

The creatinine levels in both serum and urine were detected using alkaline picric acid method under the guidance of commercial kits (Nan Jing Jian Cheng Bioengineering Institute, China). The creatinine clearance was calculated on the basis of urinary creatinine, serum creatinine, urine volume, and body weight as described previously [16]. Blood urea nitrogen level was measured using urea enzymatic colorimetric kit (Nan Jing Jian-Cheng Bioengineering Institute, China). 24-hour urinary protein was determined by the BCA method (Beyotime Institute of Biotechnology, China).

### 2.6. Determination of Kidney Histopathology

As described in details previously [17], HE staining was performed to determine kidney histopathology. The severity of renal damage was estimated by the following parameters: (1) total glomerular surface area and (2) mesangial matrix injury score expressed by mesangial surface area/glomerular total surface area.

### 2.7. Determinations of MDA, GSH, LDH, and TGF-*β*1 in the Urine

Kidney was homogenized using an ElectroMotion glass homogenizer (Ningbo Scientz Biotechnology Co., China) as describe previously [18]. After centrifugation, the supernatant was kept under −80°C before determinations of MDA, LDH, and GSH. The protocols of GSH and MDA measurements were remanded by commercial kits (Nan Jing Jian Cheng Bioengineering Institute, China). Urinary TGF-*β*1 was quantified by ELISA using commercial kits (BioSource, Camarillo, CA, USA) according to the manufacturer's instructions.

### 2.8. RT Polymerase Chain Reaction (PCR)

The protocol of RT-PCR was described previously by us [19]. In short, total RNA was extracted from each renal cortex tissue using 1 mL Trizol reagent (GIBCO, USA) according to the manufacturer's protocol. RNA concentrations were determined by the A260/280 ratio using a spectrophotometer and the quality was assessed on a 1.5% ethidium bromide-agarose gel. Absorbance ratios between 1.90 and 2.15 indicated pure RNA samples. Three micrograms of total RNA were reverse transcribed with oligo-dT primer and M-MLV reverse transcriptase (TIANGEN, China). One microgram of the reaction mixture was used in each PCR containing a pair of specific primers for rat NHE1, TGF-*β*1, and GAPDH. The sequences of the NHE1 primers specific for rats were sense 5′-CACGCTGTGGAATGCT-3′ and antisense 5′-GAAGATGTCCGAGATGC-3′. PCR product was 289 bp. Sequences of the TGF-*β*1 specific for rats were sense 5′-GCCAAGACCCTAACA-3′ and antisense 5′-CACTGAAGTCCACCAA-3′. PCR product was 381 bp. GAPDH mRNA was codetected with sense 5′-CAATGTATCCGTTGTGG-3′ and antisense 5′-GTCCAGGGTTTCTTACTC-3′. PCR product was 307 bp. Target sequences were amplified at 1°C below Tm using the same amount of cDNA for all primer sets, and the cycle number was adjusted between 30 and 35 to yield visible products within the linear amplification range. PCR products then were run on 1.5% agarose gels and photographed under ultraviolet light. Densities of bands were measured by scanning densitometry with Image J analysis system software and normalized to GAPDH in the same sample.

### 2.9. Western Blotting

As described previously [19], tissues were homogenized on ice in cell-lysis buffer (20 mM Tris-HCl, pH 7.5, 150 mM NaCl, 1 mM Na_2_EDTA, 1 mM EGTA, 1% Triton, 2.5 mM sodium pyrophosphate, 1 mM beta-glycerophosphate, 1 mM Na_3_VO_4_, and 1 *μ*g/mL leupeptin) and 1 mM PMSF. Cell was lysated with cell-lysis buffer. The protein content was assayed by BCA protein assay reagent (Pierce, USA). 20 *μ*g proteins were loaded to SDS-PAGE and then transferred to membrane. Membrane was incubated with a 1 : 1000 dilution of primary antibody, followed by a 1 : 2000 dilution of horseradish peroxidase-conjugated secondary antibody. Protein bands were visualized by ECL (GE Healthcare). The intensity (area X density) of the individual bands on Western blots was measured by densitometry (model GS-700, Imaging Densitometer; Bio-Rad). The background was subtracted from the calculated area. We used control as 100%.

### 2.10. *Ex Vivo* Experimental Designs

Renal slices were divided into 7 groups. Group 1 included the following: slices incubated with culture medium of DMEM/F12; group 2 included the following: slices incubated with culture medium containing BSA (200 *μ*g/mL); group 3 included the following: slices incubated with culture medium containing cariporide (1 *μ*M, H-car); group 4 included the following: slices incubated with culture medium containing AGEs (200 *μ*g/mL); group 5 included the following: slices incubated with culture medium containing AGEs plus cariporide (0.1 *μ*M, L-car); group 6 included the following: slices incubated with culture medium containing AGEs plus cariporide (1 *μ*M); group 7 included the following: slices incubated with culture medium containing AGEs plus antibody of AGEs receptor (5 *μ*g/mL, Ab-RAGE). Slices were incubated with these treatments for 2 hours.

### 2.11. *In Vivo *Experimental Design

SD rats were divided into 5 groups: group 1: control group; group 2: BSA-injected group; group 3: AGEs-injected group; group 4: AGEs-injected plus NAC treatment group; group 5: AGEs-injected plus cariporide treatment group. Rats in group 1 were fed with regular diet and tap water. Rats in group 2 received tail vein injection of BSA (100 mg/kg/day). Rats in group 3 received tail vein injection of AGEs (100 mg/kg/day). Rats in group 4 received tail vein injection of AGEs plus gavage with NAC (200 mg/kg/day). Rats in group 5 received tail vein injection of AGEs plus gavage with cariporide (1 mg/kg/day). The injection was performed on rats under anesthesia with diethyl ether. All treatments were performed for 12 consecutive weeks in rats fed with regular diet and tap water. No rat died during the whole experiment.

### 2.12. Statistical Analyses

All values are expressed as mean ± S.E.M. The results were carried out by one-way analysis of variance (ANOVA), followed by the Student-Newman-Keuls test for multiple comparisons with SPSS 11.5. A *p* value of 0.05 or less was considered significant.

## 3. Results

### 3.1. AGEs Time-/Dose-Dependently Induce LDH Leakage in Isolated Renal Cortex from Rats

**Figure 1:**
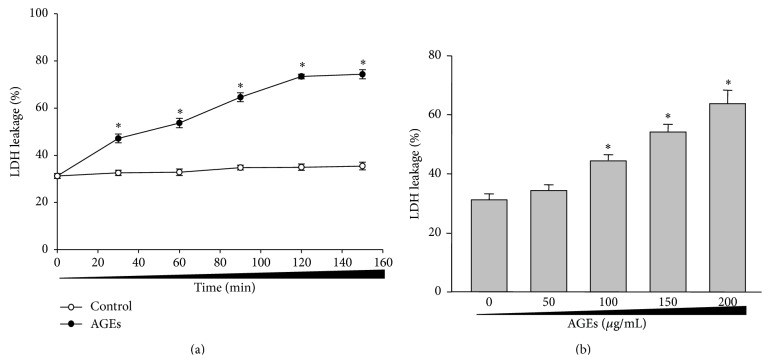
AGEs time-/dose-dependently increase LDH leakage in isolated renal cortex from rats. Cortex from isolated rat kidney was sliced into small pieces with the thickness of 0.3–0.5 mm. The slice was incubated with AGEs as indicated times and concentrations. LDH leakage was assayed in slice of renal cortex. (a) Time course of AGEs on LDH leakage. (b) Dose course of AGEs on LDH leakage. All data were expressed as mean ± SD. *N* is 5 in each group. ^*∗*^ *P* < 0.05 versus control (0).

In order to test the hypothesis, we firstly investigated whether AGEs caused renal dysfunction in rats by measuring LDH leakage in isolated renal cortex from rats, which is an indicator of loss of membrane integrity [20]. As shown in Figure 1(a), incubation of rat renal cortex slice with AGEs (100 *μ*g/mL) from 20 to 160 minutes increased LDH leakage in a time-dependent manner. BSA control had no effects on LDH leakage. Following treatment of 50–200 *μ*g/mL for 2 hours, LDH leakage significantly began to increase when the concentration of AGEs is 100 *μ*g/mL or above (Figure 1(b)). These pieces of data indicate that AGEs induce LDH leakage in isolated renal cortex, which is time/dose dependent.

### 3.2. AGEs Activates Cariporide-Sensitive NHE1 in Cultured Renal Cortex Cells

**Figure 2:**
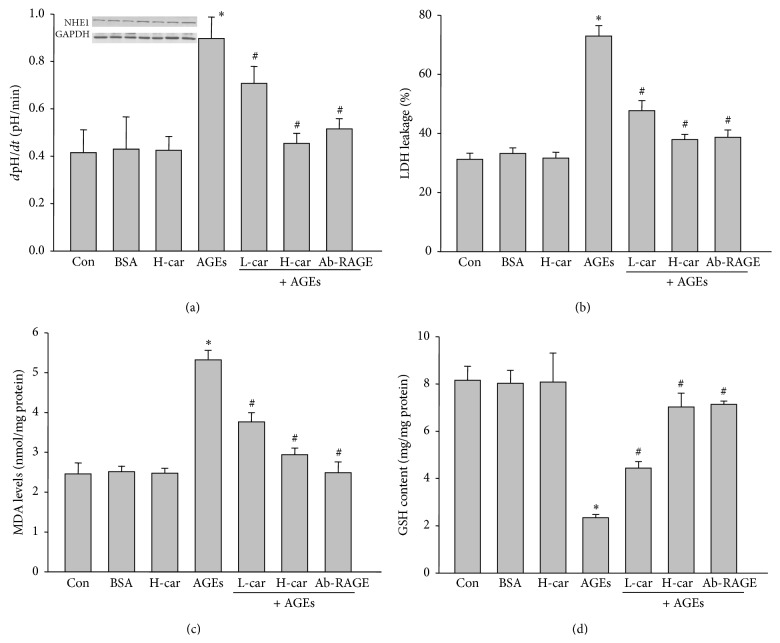
*Ex vivo* inhibition of NHE1 by cariporide reverses AGEs-increased LDH leakage in rat renal cortex. Sliced renal cortex was incubated with BSA, H-car (1 *μ*M cariporide), L-car (0.1 *μ*M cariporide), and Ab-RAGE (antibody of AGEs receptor). After treatment, (a) LDH leakage and NHE1 protein expression, (b) GSH content, (c) MAD level, and (d) NHE1 activity were measured, respectively. All data were expressed as mean ± SD. *N* is 5 in each group. ^*∗*^ *P* < 0.05 versus BSA. ^#^ *P* < 0.05 versus AGEs alone.

We next determined whether activation of NHE1 mediates AGEs-increased LDH leakage. Cariporide, a selective NHE1 inhibitor, which has been identified by us and others [7, 21], was used to inhibit NHE1 activity in this section of the present study. As indicated in Figure 2(a), AGEs, but not BSA, dramatically increased NHE1 activity in cultured renal cortex cells. Though cariporide did not inhibit NHE1 activity in cells without AGEs treatment, it significantly reduced NHE1 activity in AGEs-treated cells at low or high dose, indicating that AGEs activate NHE1, which is cariporide sensitive, consistent with our previous report [14]. The effects of cariporide on AGEs-induced NHE1 activation were mirrored by blocking receptor of AGEs by using specific antibody (Ab-RAGE), further supporting that AGEs via its receptor activate NHE1.

### 3.3. Inhibition of NHE1 by Cariporide Abolishes AGEs-Increased LDH Leakage in Renal Cortex

We then detected LDH leakage in renal cortex slice treated by AGEs. As depicted in Figure 2(b), the increased LDH leakage by AGEs was abolished by cariporide at low or high dose and Ab-RAGE, demonstrating that AGEs-induced LDH leakage enhancement is possibly related to its receptor and subsequent NHE1 activation.

### 3.4. Cariporide Normalizes the Redox State in AGEs-Treated Rat Renal Cortex

Inhibition of NHE1 has been reported to suppress oxidative stress in vascular system [22, 23]. Thus, we hypothesized that cariporide may inhibit AGEs-induced oxidative stress to maintain membrane integrity in isolated renal cortex. We evaluated the levels of oxidative stress by determinations of MDA and GSH, two markers of oxidative stress in cells [24]. In Figures 2(c) and 2(d), either cariporide or Ab-RAGE reduced the AGEs-increased MDA and AGEs-decreased GSH levels in renal cortex, suggesting that inhibition of NHE1 by cariporide normalizes the redox state in AGEs-treated rat renal cortex.

### 3.5. *In Vivo *Administration of Cariporide Inhibits AGEs-Induced Kidney Hypertrophy and Glomerular Sclerosis in AGEs-Injected Rats

AGEs are the major factors in diabetic nephropathy [25].* Ex vivo* experiments indicated the beneficial effects of cariporide on AHGEs-induced membrane disruption of renal cortex. We next investigated the* in vivo* effects of cariporide on AGEs-induced renal dysfunction. As shown in Table 1, 12-week injection of AGEs via tail vein did not affect the body weight and blood sugar levels in rats. However, AGEs dramatically increased the weight of kidney, as indicated by kidney index. Treatment of cariporide failed to alter body weight and blood glucose levels but inhibited the hypertrophy of kidney.

**Figure 3:**
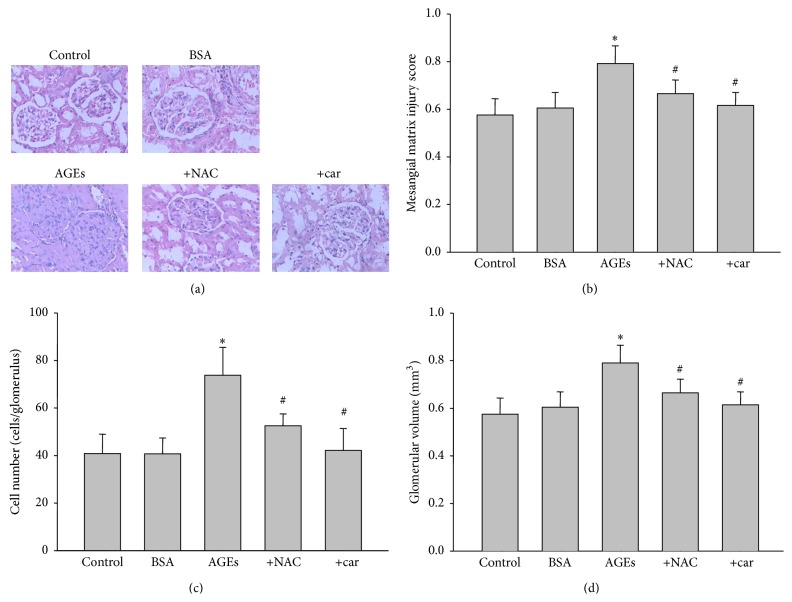
Inhibition of NHE1 improves AGEs-induced abnormality of glomerular structure in rats. The rats received a tail vein injection of AGEs (100 mg/kg) followed by treatment with or without N-acetylcysteine (200 mg/kg/day) or cariporide (1 mg/kg/day) for 12 weeks. At the end of experiments, rats were sacrificed under anesthesia. (a) Morphology of glomerular in kidney by HE staining. (b) Mesangial matrix injury score, (c) cell numbers in glomerular, and (d) glomerular volume were determined. Data are expressed as mean ± S.E.M. *N* is 10–15 in each group. ^*∗*^ *P* < 0.05 versus BSA. ^#^ *P* < 0.05 versus AGEs alone.

The hypertrophy of kidney induced by AGEs was further supported by morphological analysis by HE staining. As indicated in Figure 3(a), compared to BSA-treated rats, widespread glomerular sclerosis was observed in AGEs alone challenged rats, as well as increased mesangial matrix injury score (Figure 3(b)), cell numbers (Figure 3(c)), and glomerular volume (Figure 3(d)) by quantitative analysis. In contrast, all these effects induced by AGEs were corrected by cariporide intervention. Inhibition of oxidative stress by NAC also mimicked these effects induced by cariporide in AGEs-injected rats. Taking these data together, it suggests that inhibition of NHE1 protects kidney structure in AGEs-injected rats, which is related to suppression of oxidative stress.

### 3.6. Cariporide Inhibits Ages-Induced Renal Dysfunction in Rats

**Figure 4:**
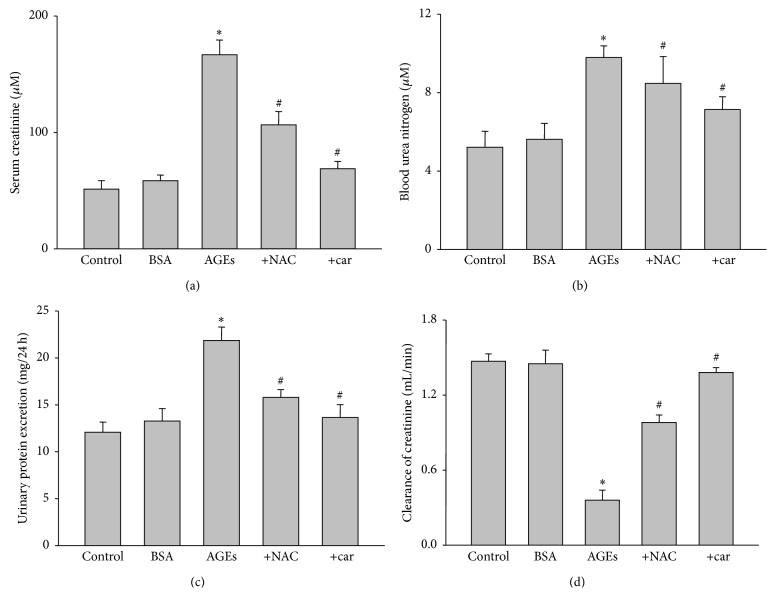
*In vivo* administration of cariporide prevents renal dysfunction in AGEs-injected rats. The rats received a tail vein injection of AGEs (100 mg/kg) followed by treatment with or without N-acetylcysteine (200 mg/kg/day) or cariporide (1 mg/kg/day) for 12 weeks. At the end of experiments, (a) serum creatinine level, (b) blood urea nitrogen, (c) urinary protein excretion, and (d) clearance of creatinine were measured, respectively. All data were expressed as mean ± SD. *N* is 5 in each group. ^*∗*^ *P* < 0.05 versus BSA. ^#^ *P* < 0.05 versus AGEs alone.

The protective effects of cariporide in kidney of AGEs-injected rat were further confirmed by analysis of renal function. Compared to BSA-injected rats, AGEs remarkably caused renal dysfunction as increased serum creatinine (Figure 4(a)), blood urea nitrogen (Figure 4(b)), and urinary albumin excretion (Figure 4(c)) and decreased clearance of creatinine (Figure 4(d)). As expected, cariporide reversed AGEs-induced enhancements of serum creatinine, blood urea nitrogen, and urinary albumin excretion and reduction of clearance of creatinine. The effects of cariporide on renal function were also copied by NAC. These data suggest that activation of NHE1 and oxidative stress are key steps contributing to AGEs-induced renal dysfunction.

### 3.7. AGEs via NHE1 Activation Induces Oxidative Stress in Rat Kidney

**Figure 5:**
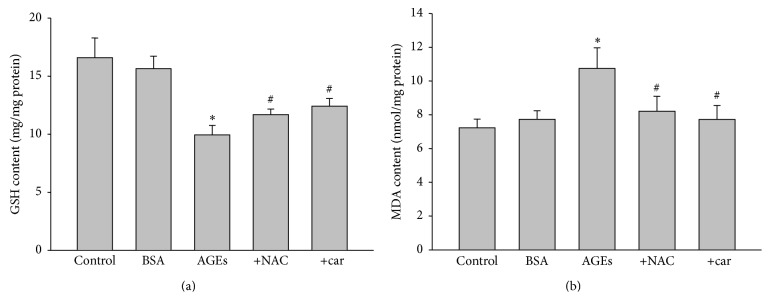
Cariporide recues AGEs-induced oxidative stress in kidney of rats. The rats received a tail vein injection of AGEs (100 mg/kg) followed by treatment with or without N-acetylcysteine (200 mg/kg/day) or cariporide (1 mg/kg/day) for 12 weeks. At the end of experiments, rats were sacrificed under anesthesia. (a) GSH content and (b) MAD level were determined. All data were expressed as mean ± SD. *N* is 10–15 in each group. ^*∗*^ *P* < 0.05 versus BSA. ^#^ *P* < 0.05 versus AGEs alone.

In order to establish the relationship between NHE1 activation and oxidative stress in AGEs-induced renal damage, we examined the levels of oxidative stress in cariporide-treated AGEs-injected rats. As indicated in Figures 5(a) and 5(b), both increased MDA and decreased GSH were induced by AGEs in rats, which were reduced by cariporide or NAC, indicating cariporide via inhibition of NHE1 suppress oxidative stress in AGEs-injected rats.

### 3.8. Cariporide Inhibits Gene Expressions of NHE1 and TGF-*β*1 in Kidneys from AGEs-Injected Rats

**Figure 6:**
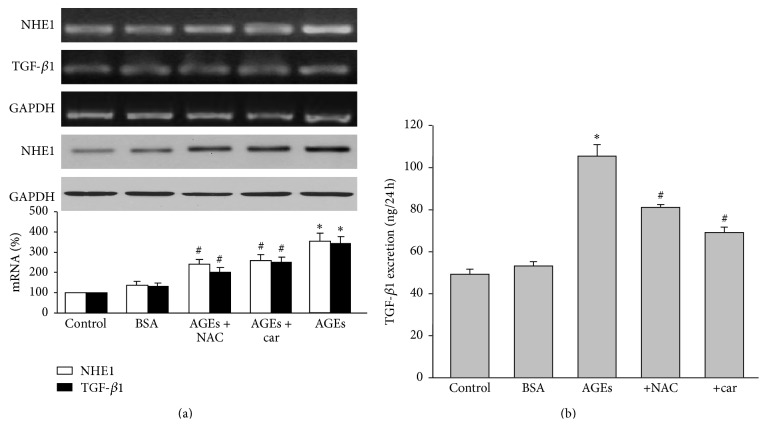
Cariporide reduces the gene expressions of NHE1 and TGF-*β*1 in renal cortex from AGEs-injected rats. The rats received a tail vein injection of AGEs (100 mg/kg) followed by treatment with or without N-acetylcysteine (200 mg/kg/day) or cariporide (1 mg/kg/day) for 12 weeks. At the end of experiments, rats were sacrificed under anesthesia. (a) mRNA levels of NHE1 and TGF-*β*1 and protein level of NHE-1 were assayed by RT-PCR or Western blot. (b) TGF-*β*1 excretion of rat urine. All data were expressed as mean ± SD. *N* is 10–15 in each group. ^*∗*^ *P* < 0.05 versus BSA. ^#^ *P* < 0.05 versus AGEs alone.

We finally determined the effects of cariporide on TGF-*β*1, which is a key mediator for diabetic nephropathy [26]. Compared with BSA-treated rats, AGEs increased NHE1 and TGF-*β*1 gene expressions, as determined by RT-PCR (Figure 6(a)). Cariporide obviously downregulated NHE1 and TGF-*β*1 gene expressions to the level of BSA-treated group. The reductions of NHE1 and TGF-*β*1 gene expressions induced by NAC were weaker than cariporide. The effects of cariporide and NAC on TGF-*β*1 gene expression were further confirmed by assaying urinary TGF-*β*1 excretion (Figure 6(b)). Collectively, it indicates that NHE1 is a potential target of cariporide to prevent renal function in AGEs-injected rats.

## 4. Discussion

The present study demonstrates that AGEs* ex vivo* or* in vivo* cause glomerular sclerosis and renal dysfunction, which is abrogated by NHE1 inhibition, blockage of AGEs receptor, and suppression of oxidative stress. Mechanistically, the detrimental effects of AGEs on kidney function might be related to activation of its receptor and sequent activation of NHE1, resulting in upregulation of oxidative stress. In this way, cariporide, a selective NHE1 inhibitor, normalizes the redox state in renal cortex and functions.

AGEs can accumulate in diverse biological settings, such as diabetes, inflammation, renal failure, and aging [27]. Many studies support that interactions between AGEs and its receptor are involved in the pathogenesis of diabetic complications, in particular, nephropathy [1, 25, 28]. The formation and accumulation of AGEs adducts in various tissues are associated with altered protein structure and function. In addition, AGEs are able to activate intracellular signaling by binding to specific receptors [29]. A number of AGEs inhibitors and crosslink breakers, such as aminoguanidine and ALT-711, have been shown to prevent the formation and break the crosslink of AGEs [30, 31]. However, they could not affect the cellular interactions of the existing AGEs with its receptor. In this present study, we injected exogenous AGEs into rats to mimic diabetic nephropathy and examined the effects of NHE1 selective inhibitor cariporide on kidney function. We observed that AGEs alone resulted in the increase of glomerular sclerosis and renal dysfunction, which are reversed by cariporide. This is the major discovery of this study.

Another important discovery is that NHE1 inhibition reduces oxidative stress induced by AGEs in diabetic nephropathy. AGEs may be linked to the increased reactive oxygen species (ROS) by decreasing antioxidative enzyme including superoxide dismutase and catalase, diminishing glutathione stores [32]. Our study indicated that AGEs significantly increased production of MDA and lower GSH content, while treatment with cariporide and NAC dramatically decreased MDA concentration and restored GSH level. Our previous study has showed that cariporide could prevent oxidative stress reaction mediated by high glucose [6, 33]. These findings confirmed that there was a close relationship between the oxidative stress and the changed activity of NHE1. During the pathologic process of AGEs in renal damage, NHE1 may be also activated by ROS due to AGE-RAGE reaction because ROS is also a well-known activator of NHE1 [34]. It should be noted that this study has been examined only in animal experiment; the precise mechanism remains to be determined in cell studies.

TGF-*β*1 is a multifunctional cytokine produced by tubular, interstitial, and glomerular cells. It stimulates the synthesis of ECM such as collagen and laminin and blocks ECM degradation through inhibition of matrix metalloproteinase [35]. It is considered to be the strongest cytokine to glomerulosclerosis in diabetic nephropathy and a last major mediator induced by a variety of damaging factors in the pathological process [36]. Therefore, we tentatively put forward that NHE1 function as a platform to link AGEs with TGF-*β*1 pathway. In this study, AGEs treated alone rats showed widespread fibrosis in renal glomerulus, parallel increase of TGF-*β*1 expression in the renal cortex, and urinary TGF-*β*1 excretion. Cariporide treatment congruously decreased the above index and prevented renal glomerulus fibrosis, suggesting that NHE1 plays a decisive role in activation of TGF-*β*1 pathway of renal injury induced by AGEs. It is worth more detailed studies in the future to further confirm the precise pathway.

In conclusion, we have identified that inhibiting NHE1 with cariporide exhibited marked protection from AGEs-mediated renal damage. NHE1 may function as a structural scaffold to link AGEs with TGF-*β*1 signaling in renal damage. These findings suggest that NHE1 is a promising target for the treatment of diabetic nephropathy and makes cariporide a promising drug for the future treatment of diabetic nephropathy.

## Figures and Tables

**Table 1:** General parameters in rats.

Groups	BG (mM)	BW (g)	KI (g/kg)
Vehicle	5.71 ± 0.59	385.81 ± 11.67	3.18 ± 0.272
BSA	5.94 ± 0.67	383.35 ± 9.59	3.41 ± 0.194
AGEs	6.07 ± 0.71	356.40 ± 5.94	5.07 ± 0.537^*∗*^
AGEs + NAC	5.29 ± 0.32	368.35 ± 4.38	3.75 ± 0.237^#^
AGEs + car	5.80 ± 0.54	373.80 ± 3.10	3.62 ± 0.142^#^

The rats received a tail vein injection of AGEs (100 mg/kg) followed by treatment with N-acetylcysteine (200 mg/kg/day) or cariporide (1 mg/kg/day). 12 weeks later, all rats were sacrificed under anesthesia and blood glucose (BG), body weight (BW), and kidney index (KI) were determined. All data were expressed as mean ± SD. *N* is 10–15 in each group. ^*∗*^ *P* < 0.05 versus BSA. ^#^ *P* < 0.05 versus AGEs alone.
